# The Neuropeptide Y Y_1_ Receptor: A Diagnostic Marker? Expression in MCF-7 Breast Cancer Cells Is Down-Regulated by Antiestrogens *In Vitro* and in Xenografts

**DOI:** 10.1371/journal.pone.0051032

**Published:** 2012-12-07

**Authors:** Martin Memminger, Max Keller, Miroslaw Lopuch, Nathalie Pop, Günther Bernhardt, Erwin von Angerer, Armin Buschauer

**Affiliations:** Department of Pharmaceutical/Medicinal Chemistry II, Institute of Pharmacy, University of Regensburg, Regensburg, Germany; Medical School of Hannover, United States of America

## Abstract

The neuropeptide Y (NPY) Y_1_ receptor (Y_1_R) has been suggested as a tumor marker for *in vivo* imaging and as a therapeutic target. In view of the assumed link between estrogen receptor (ER) and Y_1_R in mammary carcinoma and with respect to the development of new diagnostic tools, we investigated the Y_1_R protein expression in human MCF-7 cell variants differing in ER content and sensitivity against antiestrogens. ER and Y_1_R expression were quantified by radioligand binding using [^3^H]-17β-estradiol and the Y_1_R selective antagonist [^3^H]-UR-MK114, respectively. The latter was used for cellular binding studies and for autoradiography of MCF-7 xenografts. The fluorescent ligands Cy5-pNPY (universal Y_1_R, Y_2_R and Y_5_R agonist) and UR-MK22 (selective Y_1_R antagonist), as well as the selective antagonists BIBP3226 (Y_1_R), BIIE0246 (Y_2_R) and CGP71683 (Y_5_R) were used to identify the NPY receptor subtype(s) by confocal microscopy. Y_1_R functionality was determined by mobilization of intracellular Ca^2+^. Sensitivity of MCF-7 cells against antiestrogen 4-hydroxytamoxifen correlated directly with the ER content. The exclusive expression of Y_1_Rs was confirmed by confocal microscopy. The Y_1_R protein was up-regulated (100%) by 17β-estradiol (EC_50_ 20 pM) and the predominant role of ERα was demonstrated by using the ERα-selective agonist “propylpyrazole triol”. 17β-Estradiol-induced over-expression of functional Y_1_R protein was reverted by the antiestrogen fulvestrant (IC_50_ 5 nM) *in vitro*. Furthermore, tamoxifen treatment of nude mice resulted in an almost total loss of Y_1_Rs in MCF-7 xenografts. In conclusion, the value of the Y_1_R as a target for therapy and imaging in breast cancer patients may be compromised due to Y_1_R down-regulation induced by hormonal (antiestrogen) treatment.

## Introduction

Neuropeptide Y (NPY), a 36 amino acid peptide, is one of the most abundant peptides in the central and peripheral nervous system of mammals, involved in numerous (patho)physiological functions such as food intake, blood pressure, regulation of hormone secretion, anxiety and memory [1]. In humans NPY exerts its biological effects by interaction with at least four distinct G protein coupled receptors designated Y_1_ (Y_1_R), Y_2_ (Y_2_R), Y_4_ (Y_4_R), and Y_5_ (Y_5_R) [2]. The Y_1_R subtype was the first NPY binding receptor to be cloned [3]. Its constitutive expression and functionality in human erythroleukemia (HEL) cells [4] and in SK-N-MC neuroblastoma cells [5] is well established. Y_1_ and Y_2_ receptors were recently reported to be expressed in several human cancers and were therefore proposed as potential targets for diagnosis and treatment [6]–[14]. Mammary carcinomas revealed an 85% incidence of Y_1_R expression, whereas Y_2_R was shown to be the less expressed NPY receptor subtype [15]. An estrogen induced expression of Y_1_R mRNA in MCF-7 breast cancer cells was shown in a differential screening study [16]. Later, investigations confirmed the up-regulation of Y_1_R mRNA after estrogen treatment, and suggested a functional role of the Y_1_R in cell signaling and proliferation [17]. Very recently, a DOTA (1,4,7,10-tetraazacyclododecane-1,4,7–10-tetraacetic acid) substituted Y_1_R selective peptide for radiolabeling with metallo positron emitters for PET imaging of breast cancer was described [18] and the use of a Y_1_R selective ^99m^Tc-labeled peptide in whole body scintimammography was reported [11].

In consideration of the assumed link between ER and Y_1_R in breast cancer and the potential value of new diagnostic tools we combined tumorpharmacological investigations with our work on receptor subtype-selective ligands for the detection of NPY receptors. Y_1_R selective fluorescence and radiolabeled compounds, recently developed in our laboratory, as well as a set of reference substances were used as pharmacological tools. To evaluate the working hypothesis that the Y_1_R is a potential diagnostic target in breast cancer, we performed preclinical investigations on ER and NPY receptor expression and function, taking into account the impact of standard therapies using antiestrogens or aromatase inhibitors.

**Figure 1:**
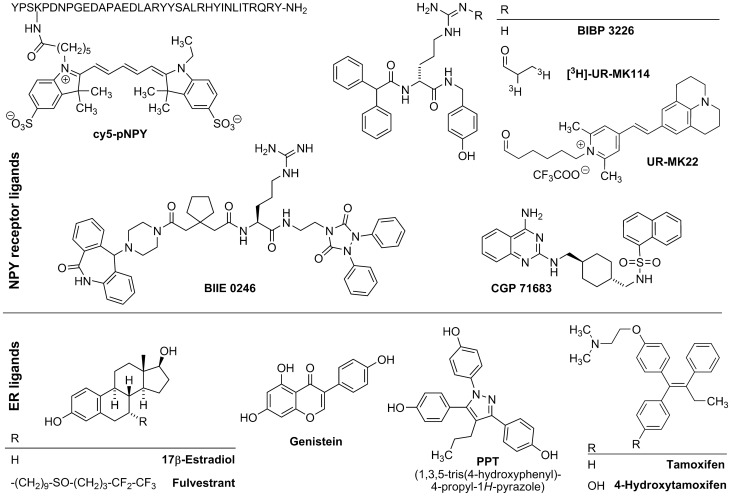
Chemical structures of the compounds used as pharmacological tools.

The recently developed highly potent and selective tritiated Y_1_R antagonist [^3^H]-UR-MK114 (Fig. 1) [19], an (*R*)*-*argininamide derived from BIBP3226 [20], was applied to quantify Y_1_R protein expression in radioligand binding assays using adherent live cells. In the present study different subclones of MCF-7 breast cancer cells with different estrogen receptor (ER) content were analyzed with respect to a correlation between ER and Y_1_R expression. Furthermore, the influence of ER agonists and antagonists on the expression of the functional Y_1_R protein was determined in a fura-2 assay. In addition to in vitro studies, the Y_1_R expression was investigated by autoradiography of MCF-7 xenografts from nude mice supplemented with 17β-estradiol on one hand, and treated with tamoxifen on the other hand.

## Materials and Methods

### Ethics Statement

Animal studies. The use of animals in this study complies with the Guide for the Care and Use of Laboratory Animals (NIH publication no. 86-23, revised 1985) and the current German law on the protection of animals. The animal experiment was approved by the Regierung der Oberpfalz (Bavaria, Germany) (document number: 54–2531.2-28/08).

Cancer cells. MCF-7 (HTB 22), MDA-MB-231, T-47-D breast cancer cells were from the American Type Culture Collection (Rockville, MD). HCC1806 and HCC1937 breast cancer cells, from the ATCC (LGC Standards, Wesel, Germany), were kindly provided by Dr. Jörg Engel (University of Würzburg, Germany). A subclone of MCF-7 cells, originating from HTB 22 (ATCC), (MCF-7 (M): medium estrogen receptor content) was kindly provided by Dr. Hauke Lilie (University of Halle, Germany).

**Figure 2:**
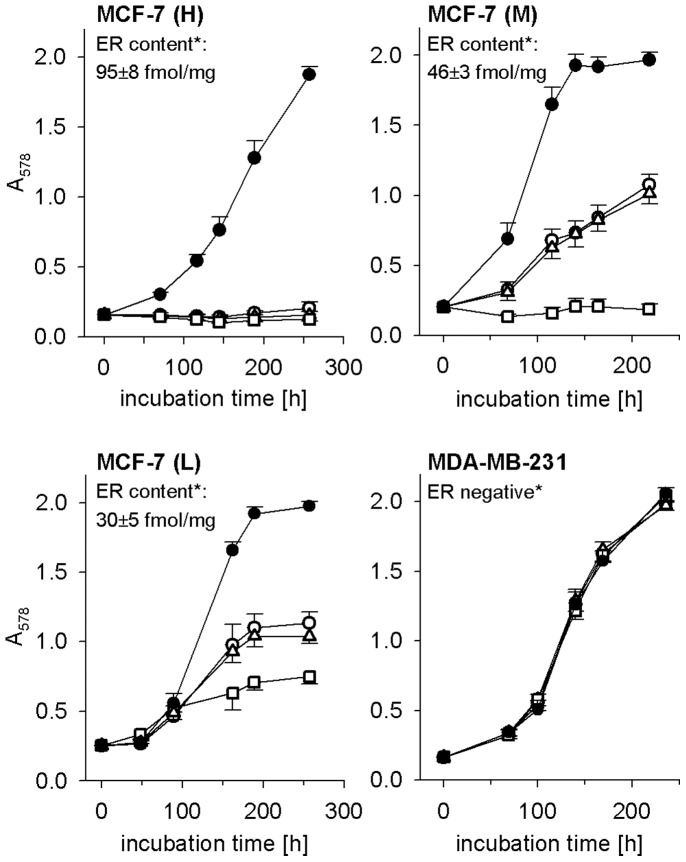
Effect of the antiestrogen 4-hydroxytamoxifen on the proliferation of breast cancer cells. Growth kinetics of three MCF-7 variants differing in ER content (high (H), medium (M) low (L)) and MDA-MB-231 (negative) breast cancer cells in the presence of 4-hydroxytamoxifen (○10 nM, Δ 100 nM, □ 1 µM) compared to vehicle (•) (ethanol at a final concentration of 0.1%). Mean values ± standard deviations (n = 16). *The ER content (mean value ± SEM, n = 3) was determined radiometrically from corresponding cytosols using [^3^H]-17β-estradiol. No specific binding was detected in the case of MDA-MB-231 cells. ER content (fmol/mg) was normalized to soluble protein.

### Materials

Fetal calf serum (FCS) was purchased from Biochrom AG (Berlin, Germany). Porcine NPY (pNPY) was kindly provided by Dr. Chiara Cabrele (Paris-Lodron-Universität, Salzburg, Austria). The Y_1_R antagonist BIBP3226 [20], the Y_1_R selective radioligand [^3^H]-UR-MK114 (a_s_ = 97 Ci/mmol) [19], the Y_2_R antagonist BIIE0246 [21], the Y_5_R antagonist CGP71683 [22], and the fluorescent cyanine-5 labeled pNPY (Cy5-pNPY) [23] were synthesized in the authors’ laboratories. 17β-Estradiol, 4-hydroxytamoxifen, Eagle minimum essential medium (EMEM), RPMI medium, and Mc Coy’s 5A medium were purchased from Sigma (Munich, Germany). [^3^H]-17β-Estradiol was from Amersham Biosciences/GE Healthcare (Freiburg, Germany). Phenol red-free Dulbecco’s minimum essential medium (DMEM) was from Invitrogen (Karlsruhe, Germany). PPT (1,3,5-tris(4-hydroxyphenyl)-4-propyl-1*H*-pyrazole, “propylpyrazole triol”) was obtained from Tocris Biosciences (Ellisville, MO). Genistein was from Roth (Karlsruhe, Germany). Fulvestrant (ICI 182.780) was a gift from Dr. M. R. Schneider (Berlin, Germany). The chemical structures of the pharmacological tools used to perform this study are summarized in Fig. 1.

**Figure 3:**
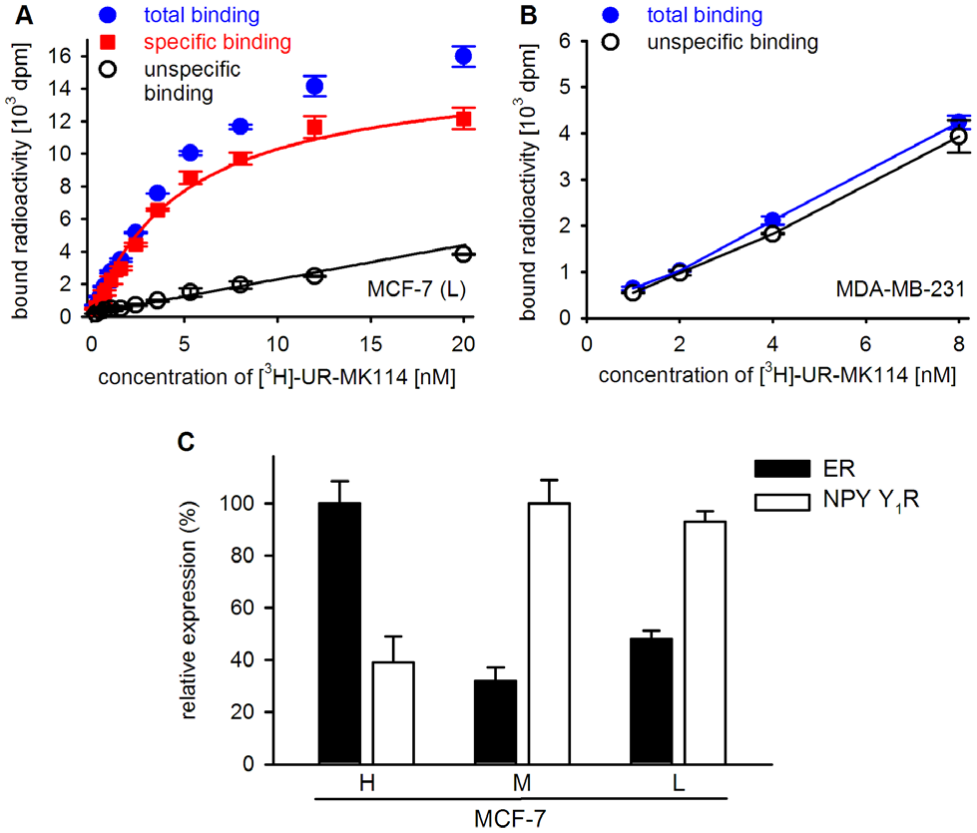
Radiochemical determination of NPY Y_1_R and ER. A,B: Basal expression of NPY Y_1_R by MCF-7 (L) and MDA-MB-231 breast cancer cells. A: Representative saturation binding curve of the Y_1_R selective tracer [^3^H]-UR-MK114 to whole MCF-7 (L) cells (K_d_ = 5 nM); values represent mean values of triplicates ± SEM; the extent of Y_1_R expression has been maintained for at least 50 passages. B: In MDA-MB-231 cells no difference between total and unspecific binding was detected; C: Comparison of the relative Y_1_R and ER expression (percent of maximum expression) in MCF-7 cell variants (H), (M) and (L) derived from radioligand binding using [^3^H]-UR-MK114 and [^3^H]-17β-estradiol, respectively.

### Cell Culture

MCF-7 cells were grown in EMEM containing 5% FCS. HCC1806, HCC1937, and T-47-D cells were cultured in RPMI medium supplemented with 10% FCS. In the case of T-47-D cells, 10 µg/L of insulin (Sigma, Munich, Germany) were supplemented. To study (anti)estrogenic effects on Y_1_R expression, the medium was replaced with EMEM (or phenol red-free DMEM) supplemented with FCS twice treated with dextran-coated charcoal (ct-FCS) [24]. MDA-MB-231 cells were cultured in Mc Coy’s 5A medium containing 5% FCS.

**Figure 4:**
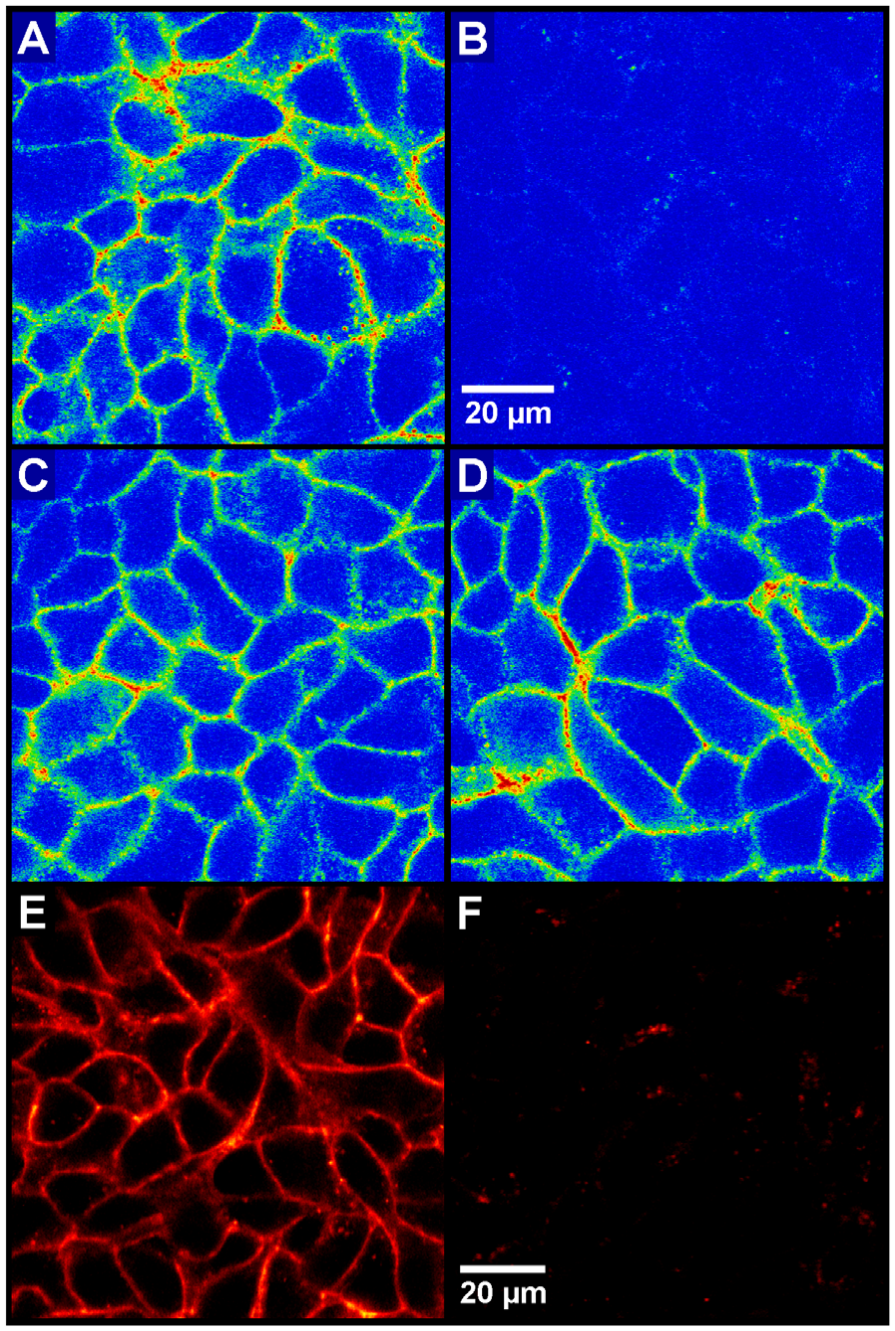
NPY Y_1_R expression in MCF-7 cells. Detection of NPY receptor subtype(s) expressed by MCF-7 (L) cells (216^th^ (A–D) and 172^th^ (E,F) passage) using confocal microscopy. A–D (rainbow): Cells were incubated with the fluorescent Y_1_, Y_2_ and Y_5_ receptor agonist Cy5-pNPY (10 nM) alone (A) or in combination with selective antagonists (Y_1_R: BIBP3226 (B), Y_2_R: BIIE0246 (C), Y_5_R: CPG71683 (D)) at a concentration of 1 µM each (100-fold excess to Cy5-pNPY). Cy5-pNPY was displaced by the Y_1_R selective antagonist only (B). E,F (glow scale): Binding of the Y_1_R selective fluorescent antagonist UR-MK22 (60 nM) to Y_1_Rs in the cell membrane. E: total binding, F: unspecific binding in the presence of BIBP3226 (100-fold excess).

### Proliferation Assay

The sensitivities of MCF-7 and MDA-MB-231 breast cancer cells against the antiestrogen 4-hydroxytamoxifen and the effect of pNPY on the growth of MCF-7 cells were determined in a previously described chemosensitivity assay [25]. Cells were grown in 96 well plates in the presence of increasing concentrations of 4-hydroxytamoxifen or pNPY, respectively. Compounds were added as 1000-fold concentrates to the respective culture media. 16 wells were processed for each compound concentration and respective vehicle control. As readout of the cell mass per well the absorbance at 578 nm was determined at different time points after staining the cells with crystal violet.

**Figure 5:**
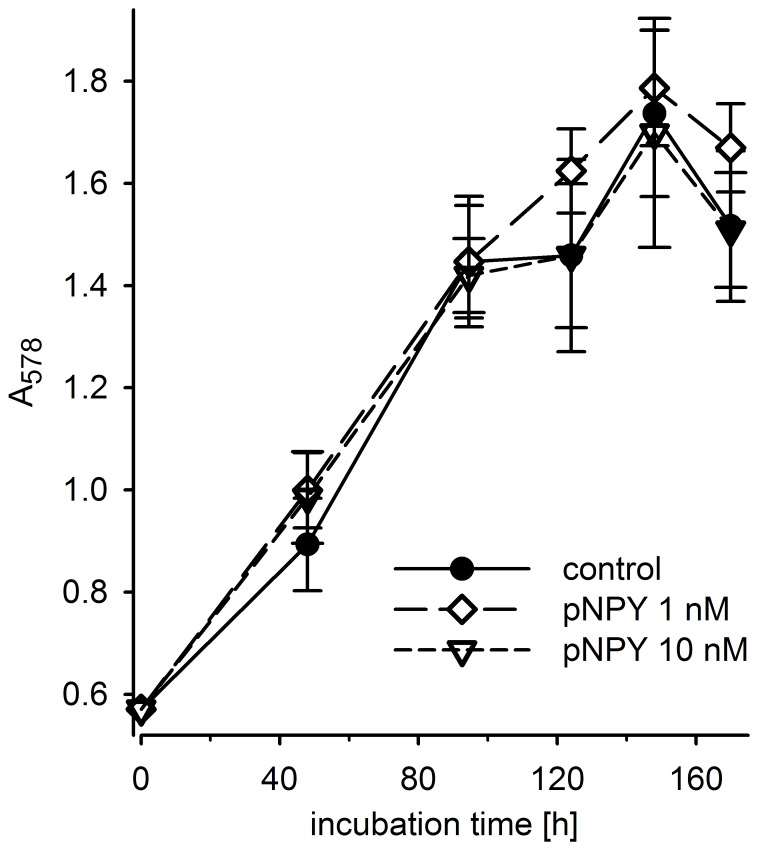
Proliferation of MCF-7 cells is unaffected by NPY. Effect of pNPY on the growth of MCF-7 (L) cells compared to the control. In all experiments the culture medium was supplemented with 17β-estradiol (1 nM).

### Cytosol Preparation

Three different MCF-7 variants (H: high ER content (wild type); M: medium ER content; L: low ER content) and MDA-MB-231 cells (ER negative) were grown in 175-cm^2^ culture flasks. When cells were confluent, the medium was removed and the cells from 8–10 flasks were harvested after trypsination. The pooled cell suspensions were centrifuged at 1200 rpm for 7 min. The pellet was washed twice with PBS and suspended in 4–5 mL of TED-Mo-buffer (10 mM Tris-HCl, pH 7.4, 10 mM Na_2_MoO_4_ (Sigma), 1 mM EDTA, 1 tablet of EDTA-free protease inhibitor cocktail (Roche, Basel, Switzerland) per 100 mL). Cells were lysed using an ultrasonic cell disrupter B15 (Branson, Danbury, CT, 3×10 cycles, 10–20 s) under ice cooling. The suspension was centrifuged for 20 min at 5000 rpm. The supernatant cell extract was decanted carefully and stored at −70°C.

**Figure 6:**
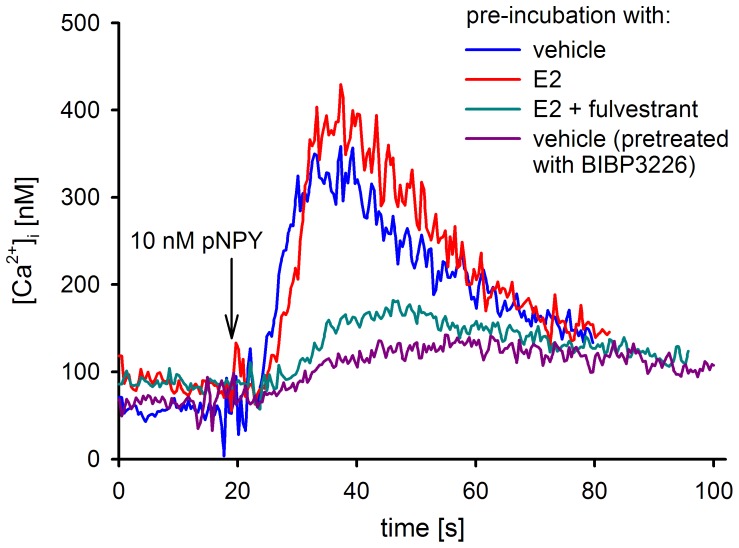
NPY Y_1_R is functionally active in MCF-7 cells. Mobilization of intracellular calcium in MCF-7 (L) breast cancer cells after stimulation with 10 nM pNPY. Calcium signals were recorded after pre-incubation of the cells with 1 nM 17β-estradiol (E2), E2 (1 nM) plus fulvestrant (100 nM) and vehicle (ethanol at a final concentration of 0.1%), respectively, for 45 hours. Cells were harvested, washed and loaded with fura-2-AM according to standard protocols. The Y_1_R selective antagonist BIBP3226 (500 nM) was added one minute before NPY stimulation.

The protein content of the cytosols was determined after appropriate dilution by Bradford’s protein assay [26] using Bradford dye reagent (BioRad Laboratories, Munich, Germany) following the manufacturer’s protocol. Absorbance was measured in a Uvikon 930 spectrophotometer (Kontron, Neufahrn, Germany) at 595 nm. A calibration curve with human serum albumin (HSA, Behringwerke, Marburg, Germany) standards was recorded to assign absorbance values to protein concentrations.

**Figure 7:**
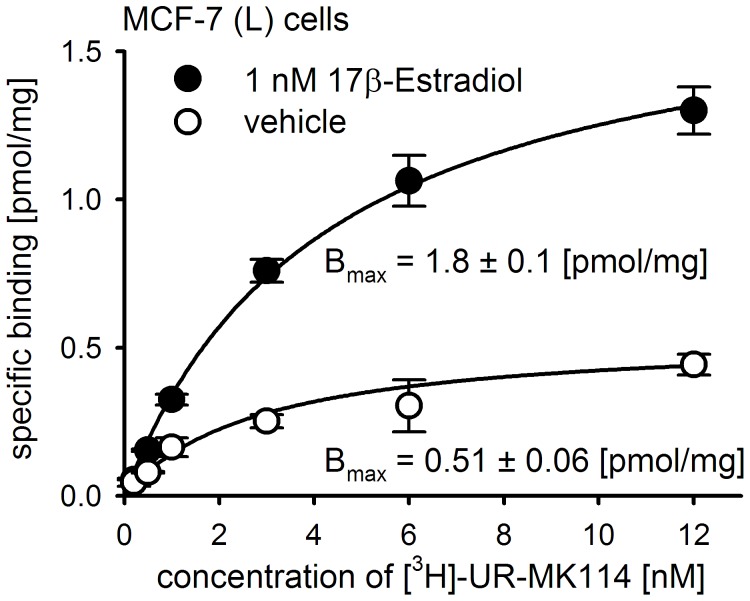
Y_1_R expression is up-regulated by estradiol. Effect of 17β-estradiol on Y_1_R expression by human breast cancer cells. Representative curves for specific binding of the Y_1_R selective radioligand [^3^H]-UR-MK114 to whole MCF-7 (L) cells after incubation with 1 nM 17β-estradiol or vehicle (ethanol at a final concentration of 0.1%) for 48 hours (n = 2). B_max_ values (fmol/mg) were normalized to protein content.

### [^3^H]-17β-Estradiol Binding Assay

The [^3^H]-17β-estradiol ([^3^H]-E2) saturation binding assay was performed in 1.5 mL-reaction vessels (Eppendorf, Hamburg, Germany) under ice cooling. Mixtures of the radioligand (added as a 5-fold concentrated solution in Tris buffer (100 µL); final concentration range: 0.1–5.0 nM) and the respective cytosol (100 µL) were diluted to a final volume of 500 µL in buffer (10 mM Tris-HCl, pH 7.4). 17β-estradiol (final concentration: 1 µM) was added to determine nonspecific binding. Total and nonspecific binding were determined in triplicate. The samples were incubated for 16–20 h at 4°C under shaking. Non-bound radioactivity was removed by the dextran-coated charcoal (DCC) method. For this purpose 0.5 mL of a an ice-cold suspension containing 0.8% charcoal (Norit A; Serva, Heidelberg, Germany) and 0.008% dextran 60 (Serva) were added to each sample, followed by incubation at 4°C for 30 min under shaking. After centrifugation (10 min at 4000 rpm), 200 µL of the supernatant were transferred into minivials containing 3 mL of liquid scintillator (Rothiszint™ eco plus; Roth, Karlsruhe, Germany). The bound radioactivity was counted in a LS6500 liquid scintillation beta counter (Beckmann Instruments, Munich, Germany).

**Figure 8:**
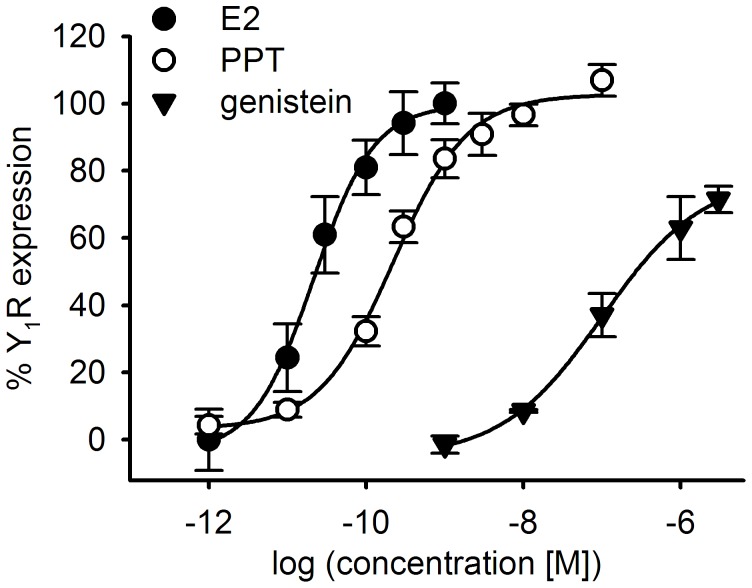
Y_1_R up-regulation is mediated by ERα. Y_1_R expression by MCF-7 (L) cells depending on stimulation with various ER agonists. The Y_1_R up-regulation induced by 1 nM 17β-estradiol (E2) was set to 100%. The Y_1_R content was determined by specific binding of [^3^H]-UR-MK114 (12 nM). E2: EC_50_ = 16±6 pM; PPT (ERα selective agonist): EC_50_ = 0.25±0.03 nM, mean values of 2 independent determinations, performed in duplicate, ± SEM; genistein: EC_50_ approximately 100 nM (single experiment, performed in duplicate).

### Whole Cell Y_1_R Radioligand Binding Assay

The maximum number of Y_1_Rs (B_max_) was determined in saturation binding experiments using the radioligand [^3^H]-UR-MK114 as previously described [19]. The average cell number per well was determined from identically processed control wells (n = 6) after counting the cells in a Neubauer improved hemocytometer.

**Figure 9:**
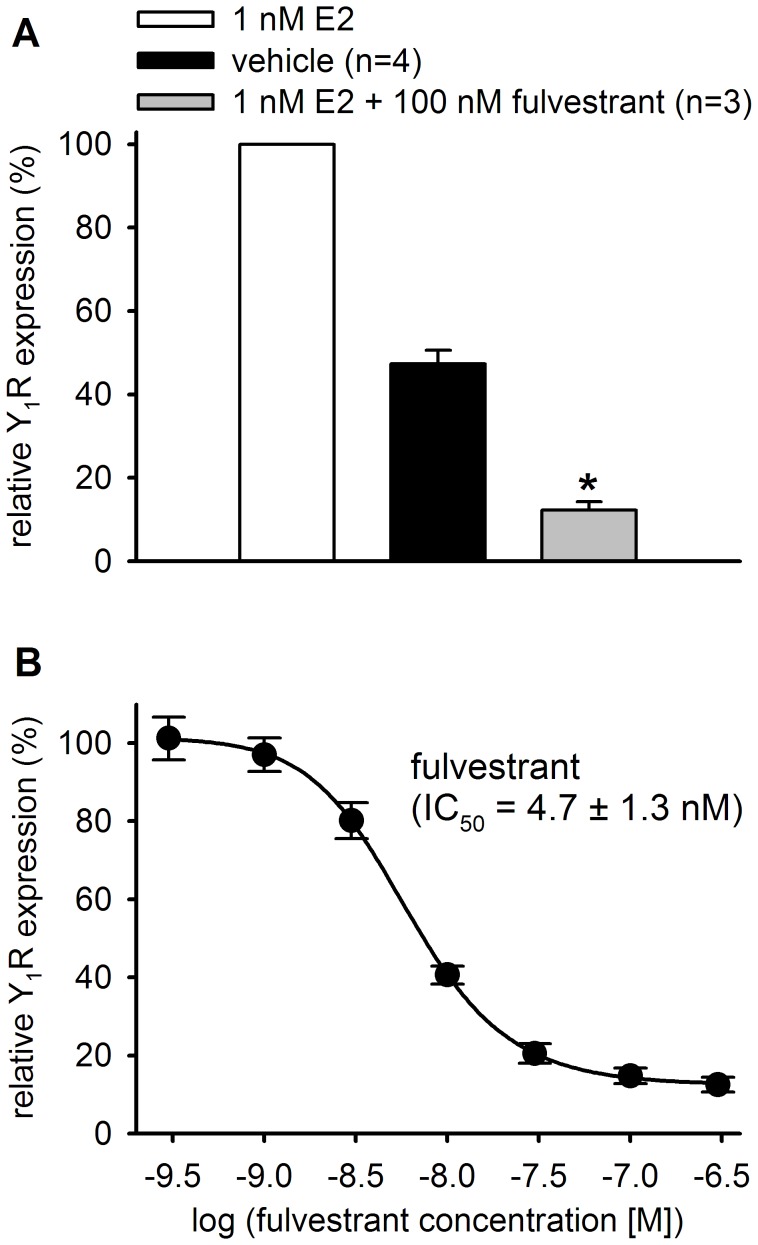
Y_1_R expression in MCF-7 cells is abrogated by antiestrogens in vitro. Effect of the pure ER antagonist fulvestrant on the estrogen stimulated Y_1_R expression in MCF-7 (L) cells. A: Inhibition of estradiol (E2, 1 nM) induced Y_1_R expression (determined with [^3^H]-UR-MK114, 12 nM) by the full ER antagonist fulvestrant. Incubation period: 48 h; basal expression: EMEM containing ct-FCS and vehicle. Mean values ± standard error of the mean (SEM); *p<0.001 compared to vehicle. B: Concentration-dependent inhibition of the estradiol (1 nM) induced Y_1_R expression by fulvestrant. The IC_50_ value ± SEM was calculated from two independent determinations performed in triplicate. The Y_1_R up-regulation induced by 1 nM 17β-estradiol (E2) was set to 100%.

For the determination of (anti)estrogenic effects on Y_1_R protein expression, MCF-7 cells were seeded in 48-well plates and grown in ct-FCS-containing medium until they had reached 70–80% confluence. 45–50 h prior to the Y_1_R binding assay, the medium was removed by suction and replaced with fresh medium (0.3 mL/well) containing the estrogens at the respective concentrations (by dilution of a 1000-fold concentrate in ethanol). For the analysis of the antagonistic effect of fulvestrant, the antiestrogen was added at multiple concentrations in the presence of 1 nM 17β-estradiol as stimulating agent. At least 6 wells per plate were processed for each (anti)estrogen concentration. All plates were prepared in duplicate as two identical sets. One set of 48 well plates was used for the Y_1_R radioligand binding assay to quantify Y_1_R expression: If not otherwise indicated, [^3^H]-UR-MK114 was added at a concentration of 12 nM with an incubation period of 20 min. From each group of replicate wells (n = 6–8), one half was used for the determination of the total binding (radioligand alone) and the other half for the determination of unspecific binding (radioligand plus 300-fold excess of pNPY). In order to exclude dissociation of the radioligand [^3^H]-UR-MK114 during the washing steps after incubation, additional experiments were performed with respect to the time period and the number of washing cycles (cf. Fig. S1).

**Figure 10:**
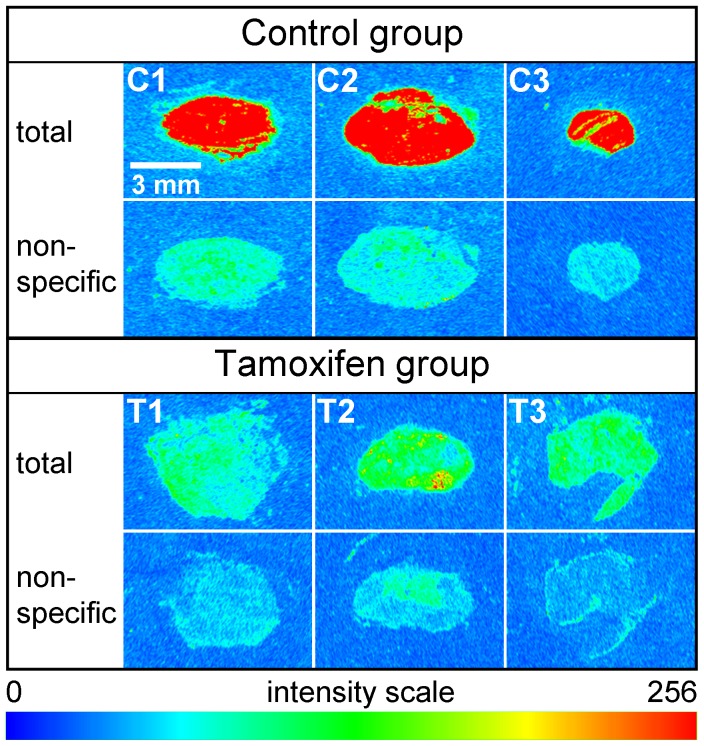
Y_1_R expression in MCF-7 xenografts is down-regulated by antiestrogens in vivo. Effect of estradiol and tamoxifen on Y_1_R expression by MCF-7 (L) xenografts in vivo determined by autoradiography using the selective Y_1_R antagonist [^3^H]-UR-MK114 (3 nM). Subcutaneously grown tumors from NMRI (nu/nu) mice bearing subcutaneous 17β-estradiol depots. The control group (3 mice, C1–C3) was treated with the vehicle (PEG400/1.8% NaCl, 1∶1). Tamoxifen group (3 mice, T1–T3): A cumulative dose of tamoxifen citrate (36 mg/kg, dissolved in PEG400/1.8% NaCl, 1∶1, at a concentration of 2.4 mg/mL) was administered by injecting three times (on day 2, 6 and 10 after explantation of the estrogen depots) 12 mg/kg subcutaneously.

The second set of plates was used as control to normalize the specifically bound radioactivity to the protein content. For this purpose, the cells of the control wells were lysed with a buffer (50–100 µL, volume dependent on the protein concentration), consisting of 25 mM Tricine (pH 7.8), 10% glycerol, 1% Triton™ X-100 (Serva) and 1 mM dithiothreitol (Sigma), for 30 min under shaking. 5 µL of each lysate were analyzed by the Bradford protein assay after appropriate dilution.

### Confocal Microscopy

Images were acquired with a Zeiss Axiovert 200 M microscope equipped with the LSM 510 laser scanner. Two days before the experiment MCF-7 (L) cells were trypsinized and seeded in ibiTreat µ-slide 8-well cover glasses (Ibidi, Planegg, Germany) in EMEM containing 1 nM 17-β-estradiol and 5% FCS. At a confluence of the cells of about 80% the culture medium was removed, the cells were washed with Leibowitz L15 culture medium (200 µL) and covered with L15 medium (100 µL) and Cy5-pNPY (100 µL of a two-fold concentrated solution in L15 medium) for total binding as well as with the competing agent (100 µL of a two-fold concentrated solution in L15 medium) and Cy5-pNPY (100 µL of a two-fold concentrated solution in L15 medium) for displacement. Images were acquired after an incubation period of 7–9 min (excitation at 633 nm (10% laser transmission), 650 nm long-pass filter).

Visualization of Y_1_Rs using the fluorescent Y_1_R-selective ligand UR-MK22 was performed as reported [27] with the following variations: on the day of the experiment confluence of the cells was about 70–80%. Images were acquired after an incubation period of 16 min (excitation at 488 nm (5.1% laser transmission), 560 nm long-pass filter).

### Calcium Assay

The intracellular Ca^2+^ concentration in MCF-7 (L) cells was measured by a spectrofluorimetric assay with the fluorescent Ca^2+^ indicator fura-2. The assay was performed by analogy with a protocol established for HEL cells in our laboratory [28]. Prior to the assay, MCF-7 cells were incubated with 1 nM 17β-estradiol alone or in combination with 100 nM fulvestrant, or the respective vehicle, for 45 h. Calcium mobilization in MCF-7 cells was stimulated by 10 nM pNPY. To antagonize the Y_1_R mediated calcium mobilization, BIBP3226 (100 nM) was added 1 min prior to the addition of pNPY. The ratio R of fluorescence intensities at 510 nm after excitation at 340 and 380 nm was used for the calculation of the calcium concentration according to the Grynkiewicz equation [29]: [Ca^2+^] = K_D_ · (R - R_min_)/(R_max_ - R) · SFB (K_D_: dissociation constant of the fura-2-Ca^2+^ complex = 224 nM; R_max_: fluorescence ratio in presence of saturating Ca^2+^ concentration (determined after the addition of 10 µL of digitonin solution (2% in water; Sigma), which caused lysis of the cells); R_min_: ratio in absence of free Ca^2+^, caused by addition of 50 µL of EGTA solution (600 mM in 1 M Tris buffer, pH 8.7) to lysed cells; SFB: correction factor; ratio of the fluorescence intensity (λ_ex_ = 380 nm, λ_em_ = 510 nm) of the Ca^2+^ free and Ca^2+^ saturated dye.

### Autoradiography

About 4 million MCF-7 (L) cells (173^rd^ in vitro passage, suspended in 0.1 µL of PBS) were subcutaneously injected into 12 female NMRI (nu/nu) mice bearing subcutaneous 17ß-estradiol depots [30] (implanted 14 days before). After 4 weeks of tumor growth, 6 animals, bearing tumors of comparable size (mean tumor area about 7×6 mm), were selected for control (3 mice) and tamoxifen treatment (3 mice). In case of the tamoxifen group, estrogen depots were explanted prior to tamoxifen administration. Tamoxifen citrate (12 mg/kg, dissolved in PEG400/1.8% NaCl 1∶1 at a concentration of 2.4 mg/mL) was injected subcutaneously on day 2, 6 and 10. The control group was treated with the vehicle. 14 days after removal of the estrogen depots, tumors were excised, immediately frozen in Tissue-Tek and stored at −78°C. Cryosections (12 µm) were obtained at −16°C with a 2800 Frigocut E freezing microtome (Reichert-Jung/Leica, Germany). Adjacent sections were mounted on three microscopic slides (Superfrost Plus, 75×25×1 mm) and kept in a chamber of 100% humidity for 1–2 min. Two slides were used to determine total and non-specific binding, and the third slide immersed in an alcoholic formaldehyde fixative (37% (w/w) formaldehyde (40 mL), 95% (v/v) ethanol (360 mL) and calcium acetate (0.2 g)) for 20 s. For total binding the sections were covered with binding buffer (ca. 800 to 1000 µL) containing [^3^H]-UR-MK114 (3 nM), and for unspecific binding with binding buffer, containing the radioligand (3 nM), pNPY (300 nM) and BIBP3226 (30 nM). The sections were incubated at room temperature (22–25°C) for a period of 8 min. After incubation, the binding buffer was removed, the slides immersed three times into ice-cold buffer split to 3 vessels (each 10 s) and finally immersed into ice-cold demineralised water (3 s). The slides were put uprightly on a paper towel for 1 min and then dried in horizontal position in a desiccator over P_4_O_10_. The slides were set in close contact with a tritium sensitive screen (PerkinElmer, 192×125 mm) using an X-ray film cassette and stored in a dark room for 15 d. The autoradiographic image was generated from the tritium screen using an imager (Cyclone Storage Phosphor System, Packard).

The fixed sections were stained according to Masson-Goldner (Jerusalem’s modification) using Weigert’s iron-haematein (45 s), rinsing (H_2_O_demin_), running tap water (10 min), differentiation with 200 mL of H_2_O_demin_ +20 mL of 2 M aq. hydrochloric acid (15 s), running tap water (10 min), rinsing (H_2_O_demin_), 0.5% aq. phosphotungstic acid (15 s), running H_2_O_demin_ (10 min), acid fuchsine-Ponceau (30 s), 1% aq. acetic acid (3×immersion), phosphoric acid-Orange G (5 s), 1% aq. acetic acid (3×immersion), 0.2% light green (3.5 min), 1% aq. acetic acid (3×immersion), 96% aq. ethanol (2×3 min), 100% ethanol (2×3 min), 100% xylene (3 min). Entellan (Merck) was used for covering.

### Data Analysis

EC_50_ (effective concentration leading to 50% induction of an effect), IC_50_ (inhibitor concentration leading to 50% inhibition of an effect) as well as B_max_ (max. number of specific binding sites) and K_D_ values were determined by Sigma Plot Software Version 9.0 (Systat Software inc., Chicago, IL) using 4 parameter sigmoid and one site saturation binding fits, respectively. To calculate the number of receptors per cell, the B_max_ value was divided by the mean cell number of six identically treated control wells. For the determination of (anti)estrogenic effects on Y_1_R expression, all mean values of specific binding (dpm/well) were normalized to the mean protein content (mg/well) and are given as percentage of the 17β-estradiol (1 nM) treated controls. Errors of calculated values determined by multiple parameters were estimated according to the Gaussian law of errors. Statistical significance was tested by Student’s t-test. P<0.05 was accepted as statistically significant.

## Results

### ER Status, NPY Y_1_R Protein Expression and Antiestrogen Sensitivity of Breast Cancer Cells

ER positive (MCF-7 subclones (H), (M), (L); T-47-D: low ER expression, 14 fmol/mg [30]) and negative (MDA-MB-231, HCC1806 and HCC1937) breast cancer cell lines were characterized in terms of antiestrogen sensitivity, ER and Y_1_R expression. Irrespective of the mean ER content, receptor expression in the individual cells of the different subclone populations is very heterogeneous (cf. Fig. S2). In Fig. 2 growth kinetics of MCF-7 subclones MCF-7 (H), MCF-7 (M) and MCF-7 (L) are compared to ER negative MDA-MB-231 cells. The MCF-7 subclones (M) and (L) show considerably decreased sensitivity against 4-hydroxytamoxifen treatment compared to the wild type (MCF-7 (H)), whereas MDA-MB-231 cells were insensitive. The sensitivity directly correlates with the ER content (cf. Fig. 2; MCF-7 (H): 95, (M): 45; (L): 30 fmol/mg protein).

The recently developed high-affinity Y_1_R selective radioligand [^3^H]-UR-MK114 was used for the detection of Y_1_Rs in saturation binding assays on living cells. Typical curves of specific and unspecific binding of [^3^H]-UR-MK114 to MCF-7 (L) cells are shown in Fig. 3A. [^3^H]-UR-MK114 revealed no Y_1_R specific binding sites in ER negative MDA-MB-231 (Fig. 3B), HCC1806 and HCC1937 (data not shown) breast cancer cells.

Fig. 3C shows the relative basal expression of Y_1_R and ER in the three investigated MCF-7 variants. Under identical culture conditions Y_1_R expression in MCF-7 (M) and MCF-7 (L) cells (91,000±4,000 and 98,000±9,000 sites/cell, respectively) was by more than a factor of two higher compared to the wild type (H) of the MCF-7 breast cancer cells (38,000±10,000 sites/cell). From the phenotypical point of view, basal Y_1_R expression is inversely associated with basal ER expression. However, this does not reflect a functional correlation due to lacking agonist stimulation of both receptors.

The expression profile of NPY receptor subtypes in MCF-7 (L) cells was investigated by confocal laser scanning microscopy using fluorescent Cy5-pNPY [23], a universal ligand with comparable affinity (K_i_ ≤ 6 nM) at the Y_1_R, Y_2_R and Y_5_R (Fig. 4A–D). Cy5-pNPY (10 nM) was totally displaced by the Y_1_R selective antagonist BIBP3226 (1 µM), but neither by the Y_2_R selective antagonist BIIE0246 [21] (1 µM) nor by the Y_5_R selective antagonist CPG71683 [22] (1 µM). The displacement of Cy5-pNPY from Y_2_R and Y_5_R by BIBP3226 (1 µM) can be excluded due to high Y_1_R selectivity (K_i_ values for Y_2_R and Y_5_R >40 µM [31]–[33]). Moreover, the sole expression of the Y_1_R was confirmed by the binding of the selective fluorescent Y_1_R antagonist UR-MK22 (Fig. 4E–F).

### Effect of NPY on MCF-7 Cell Proliferation and ER Function

As the effect of NPY on tumor cell growth is controversially discussed in the literature [8], the influence of NPY on the growth of MCF-7 cells with particularly high Y_1_ receptor status (tamoxifen low sensitive subclone (L)) was investigated in the kinetic chemosensitivity assay. As shown in Fig. 5, pNPY had no effect on the growth of this MCF-7 subclone when applied at concentrations up to 10 nM in the presence of 1 nM estradiol. A similar result was obtained in the absence of estradiol (data not shown). In a luciferase assay under the control of the ER responsive element [34] there was no unambiguous effect of NPY on the estrogenic activity of 17β-estradiol (cf. Fig. S3).

### NPY Stimulated Mobilization of Intracellular Ca^2+^ in MCF-7 Cells

To confirm the functionality of the Y_1_R expressed in MCF-7 (L) breast cancer cells in the absence and presence of ER stimulation, the coupling of the receptor to the calcium signaling cascade was investigated by a fura-2 assay. pNPY at a concentration of 10 nM induced an increase in the intracellular calcium level by a factor of four (Fig. 6). In the presence of the Y_1_R antagonist BIBP3226 (100 nM) the signal was depressed by ≈ 80%, showing the Y_1_R specificity of the signaling. The calcium response was not affected, when cells were pretreated with 17β-estradiol (45 hours), but significantly decreased after pre-incubation of the cells with fulvestrant for 45 hours (Fig. 6).

### Estrogen-dependent Expression of the Y_1_R Protein

To investigate, if estrogen receptor mediated up-regulation of Y_1_R mRNA in MCF-7 breast cancer cells reported by Amlal *et al*. [17] is paralleled at the protein level, the selective Y_1_R radioligand [^3^H]-UR-MK114 was used for binding studies. Fig. 7A shows representative saturation binding curves for the specific binding of the radioligand to MCF-7 (L) cells pretreated with 17β-estradiol (1 nM) for 48 h or its vehicle. An increase in Y_1_R protein expression by approximately 250% was observed for the estrogen pre-incubated cells (B_max_ = 1.8 and 0.51 fmol/mg, resp.) The ratio of Y_1_Rs in estrogen treated vs. untreated cells was not significantly increased when the time of incubation was prolonged to 72 hours (data not shown). Consequently, 45 to 50 hours were considered as an appropriate incubation period for the treatment of MCF-7 cells with (anti)estrogens in all following experiments. In T-47-D breast cancer cells an up-regulation of the Y_1_R after estrogen treatment occurred as well, but the expression was about 20-fold lower compared to MCF-7 (L) cells (data not shown).

To facilitate the analysis of Y_1_R regulation, the specifically bound radioactivity at a radioligand concentration of 12 nM was compared, whereupon the expression levels are presented as percentage of the control (cells treated with 1 nM 17β-estradiol). At this radioligand concentration, the saturation curves reveal an approximation of the specifically bound radioactivity to the B_max_ value (cf. Fig. 3A).

The pH indicator phenol red was reported to bring along contaminants with weak estrogenic activity [35] and might therefore contribute to basal Y_1_R expression. However, the basal Y_1_R expression was not significantly different, when cells were maintained in phenol red-free DMEM and phenol red containing EMEM, respectively (Fig. S4).

### Y_1_R up- and Down-regulation by ER Agonists and Antagonists

Fig. 8 shows concentration–response curves for the Y_1_R up-regulation by a selection of ER agonists. 17β-estradiol was applied at picomolar to nanomolar concentrations, showing a sigmoidal concentration–response relationship with an EC_50_ value of approximately 0.02 nM. Maximum Y_1_R up-regulation was achieved at a 17β-estradiol concentration of 0.5 nM (there was no further increase at concentrations of 10 and 50 nM; data not shown). PPT, an agonist with 400-fold selectivity for ERα over estrogen receptor β (ERβ) [36], was applied to demonstrate the ERα subtype dependence of Y_1_R up-regulation. The compound showed an EC_50_ value of 0.25 nM and 100% intrinsic activity compared to 17β-estradiol (Fig. 8). The non-selective, but ERβ-preferring phytoestrogen genistein up-regulated the Y_1_R protein to 70% compared to the maximum effect of 17β-estradiol. The EC_50_ value was approximately 100 nM (Fig. 8).

As depicted in Fig. 9A, the pure ER antagonist fulvestrant significantly down-regulated the Y_1_R expression below the basal expression level when co-incubated with 17β-estradiol. Fulvestrant inhibited the estradiol (1 nM) induced Y_1_R expression in a concentration-dependent manner with an IC_50_ value of approximately 5 nM (Fig. 9B). To exclude adulterations of the determined Y_1_R expression due to anti-proliferative effects of antiestrogens or growth-stimulating effects of estrogenic agents, all specific binding values were normalized to the total protein content derived from an independently conducted protein assay (Bradford).

Complementary to these in vitro experiments the Y_1_R expression was studied by autoradiography in nude mice bearing MCF-7 (L) xenografts. As obvious from Fig. 10 the subcutaneously grown human breast cancer (control, C1–C3 in Fig. 10) demonstrated high specific binding of the Y_1_R selective antagonist [^3^H]-UR-MK114. By contrast, the Y_1_R radioligand binding was extremely reduced in tumors (T1–T3) of tamoxifen treated mice. This is in agreement with Y_1_R down-regulation, because the histological grading corresponds to well differentiated adenocarcinomas of comparable size irrespective of tamoxifen treatment (histology cf. Fig. S5).

## Discussion

NPY Y_1_ and Y_2_ receptors are reported to be expressed by various malignant tumors [8], [15], [37]–[39]. The majority (85%) of human primary mammary carcinomas express the Y_1_R, whereas the Y_2_R is predominant in normal breast tissue [15]. More than 70% of breast cancers are classified as ER-positive [40] and estrogen-induced up-regulation of Y_1_R mRNA was reported previously [16], [17]. Although the role of NPY receptors in tumor biology is a matter of debate [8], the Y_1_R has been considered as a diagnostic and therapeutic target. In view of the potential value of new diagnostic tools such as the recently reported Y_1_R selective ^99m^Tc-labeled peptide [11], we performed preclinical investigations on the expression of Y_1_Rs and ERs in breast cancer cells and tumors using well-established ER and NPY receptor agonists and antagonists. In particular, the influence of estrogens and antiestrogens on the expression and function of the Y_1_R protein was studied to explore the Y_1_R as a diagnostic target considering ER status and the impact of hormonal therapy with antiestrogens or aromatase inhibitors.

Among the investigated breast cancer cell types (ER-positive: three variants of MCF-7 cells, T-47-D cells; ER-negative: MDA-MB-231 cells and the triple-negative HCC1806 and HCC1937 cells), NPY receptors were only detected in ER-positive cells (Fig. 3 and 7) and identified as the Y_1_R subtype by confocal microscopy (Fig. 4) and radioligand binding (Fig. 3 and 7). With approximately 40,000 receptors per cell, the basal Y_1_R protein density in wild type MCF-7 cells was found to be in the same range as in SK-N-MC neuroblastoma cells [19], [41]. The Y_1_R protein expression was up-regulated by treatment with 1 nM 17β-estradiol in MCF-7 and - at a lower basal level - in T-47-D breast cancer cells. The estrogen induced Y_1_R protein expression reached its maximum after two days, which is indicative of a genomic process. The basal Y_1_R level in MCF-7 cells was 40–50% of that of the 17β-estradiol treated control when grown in medium containing hormone-depleted serum (ct-FCS) (Fig. 7B). Contrary to a previous finding [17], an effect of phenol red contaminants on Y_1_R expression was excluded by comparing the basal Y_1_R expression of MCF-7 cells grown in a phenol red containing and a phenol red-free medium, respectively (Fig. 7B). The Y_1_R expression was significantly down-regulated by fulvestrant, a full ER antagonist described both, as an ER down-regulator [42] and an ER degrader [43], to approximately 25% of the basal level (Fig. 9A). As no estrogenic compounds were present in the medium supplement (ct-FCS), a ligand-independent ER activation mechanism may be involved to some extent in the basal Y_1_R expression. Ligand independent ER activation can be mediated by cross-talk activation pathways including protein kinase A and C or growth factor mediated signals [44]. In previous studies full ER antagonists such as fulvestrant were shown to be capable of blocking such signaling pathways [44].

The high expression and functionality of the Y_1_R supports speculations on a role of NPY in tumor growth, as suggested, for instance, for SK-N-MC [15], [45] and MCF-7 cells [17]. Although the Y_1_R was demonstrated to be functionally active in MCF-7 cells (Fig. 6), NPY had no effect on cell proliferation (Fig. 5), which is in accordance with very recent results on human NCI-H295R adrenocortical carcinoma cells [46].

Y_1_R expression was stimulated by 17β-estradiol in a concentration-dependent manner (Fig. 8); the EC_50_ value amounted to 20 pM. This is the first time that an up-regulation of the Y_1_R at physiologically relevant concentrations of 17β-estradiol has been demonstrated at the protein level. These results are in accordance with the work of Amlal *et al*. [17], reporting an elevation of Y_1_R mRNA expression albeit at supra-physiological estradiol concentrations (10 and 100 nM). The EC_50_ value of estradiol determined in the present study via Y_1_R up-regulation is in the same range as that reported for the up-regulation of the progesterone receptor mRNA in MCF-7 cells (44 pM; cf. [47]). As subtype selective ER antagonists are not available, appropriate agonists were used as pharmacological tools to identify the ER subtype involved. The high efficacy and potency of PPT suggests a predominant role of ERα in Y_1_R regulation, as PPT is devoid of any activity at ERβ [36]. The EC_50_ value is in good agreement with that reported for ERα from a co-transfection assay (≈ 0.1 nM, cf. [36]). Genistein, a phytoestrogen, was previously reported to be an ERβ-preferring partial (50%) agonist and a weak full ERα agonist [48]. Genistein up-regulated the Y_1_R by 70% with an EC_50_ value of 100 nM. This result matches with the reported data for ERα rather than for ERβ, underlining that Y_1_R induction is ERα mediated.

The pure antiestrogen fulvestrant inhibited the ER-stimulated Y_1_R expression in a concentration-dependent manner (Fig. 9). The IC_50_ value of 4.7 nM obtained for fulvestrant is in excellent accordance with data from a luciferase gene reporter assay [49]. Thus, the ERα-regulated expression of the Y_1_R is a suitable readout for the characterization of estrogens and antiestrogens.

The above-discussed results suggest a possible value of the Y_1_R as a surrogate marker of the ER status in breast cancer. Moreover, receptors of regulatory peptides such as NPY are in the focus of approaches to tumor targeting and molecular imaging of cancer [6]–[11], [13], [14], [18]. Therefore, we investigated the effect of estradiol and tamoxifen treatment on the Y_1_R level in MCF-7 tumors growing subcutaneously in nude mice (Fig. 10). The regimen of antiestrogen treatment (cumulative dose of 36 mg/kg tamoxifen citrate) was adjusted over 14 days (three injections, 12 mg/kg), on one hand to stop tumor growth and on the other hand to prevent tumor regression and necrosis (cf. histology, Fig. S5). In accordance with the in vitro results autoradiography of the xenografts revealed strong expression of the Y_1_R in the presence of estradiol and an almost total down-regulation after antiestrogen treatment. Y_1_R protein expression in MCF-7 cells depends on the activation state of the ER. By analogy with these findings, very recently, fulvestrant treatment was reported to down-regulate the progesterone receptor levels, monitored by PET in STAT1-deficient mammary tumors in mice [50], reflecting the response to endocrine therapy. In principle, a decrease in the expression of a membrane protein such as the Y_1_R might be exploited as a marker for the response to hormonal treatment as well. However, measuring a decrease, finally resulting in the lack of the signal is an unfavorable analytical parameter in view of high probability of false negative results. As Y_1_R down-regulation was a relatively fast process in vitro (<48 h) as well as in nude mice (<14 d) before tumor regression, negative PET results might be misinterpreted.

In conclusion, in view of the loss of the Y_1_R during tamoxifen treatment the suitability of this peptide receptor as a target for tumor therapy and imaging should be re-considered. In particular, in breast cancer patients the diagnostic value of the Y_1_R may be compromised due to Y_1_R down-regulation induced by therapeutically administered antiestrogens.

## Dedication

Dedicated to Prof. Dr. Dr. Wolfgang Wiegrebe, Regensburg, on the occasion of his 80^th^ birthday.

## Supporting Information

Figure S1 **Specific binding of [^3^H]-UR-MK114 in dpm after varying washing conditions.** (A) twice 20, 40, 60 and 90 s and (B) 2×, 3×, 4× and 5× 20 s; means ± S.E.M, n = 6. The experiments were performed to check for the dissociation of [^3^H]-UR-MK114 under the washing conditions applied in the radioligand binding assay. Basically, the experiments were conducted as already described in this paper and in [19]. Total binding was assessed with 12 nM of [^3^H]-UR-MK114, unspecific binding with radioligand (12 nM) plus a 300-fold excess of pNPY, all after an incubation time of 20 min at room temperature. A standard washing procedure of twice 20 s with ice cold buffer was set as reference. Then, conditions were varied in time and cycles, i.e. washing occurred at twice 40, 60 and 90 s as well as 3 times, 4 times and 5 times 20 s, all with n = 6. Under all washing conditions the specific binding was stable and only a negligible drop was observed with the longest period or the highest cycles.
(TIF)

Figure S2 **Immunocytochemical detection of the ERα expressed in different MCF-7 breast cancer cell variants according to the peroxidise/antiperoxidase method after paraformaldehyde fixation.** Primary anti-human ER antibody clone 6F11 (LifeSpan BioSciences, Seattle, USA) using Ventana immunostainer (Ventana Medical Systems, Tucson, USA). MCF-7 cell with (A) high, (B) medium, and (C) low ER expression.
(TIF)

Figure S3 **Effect of pNPY on the relative estrogenic activity of 17β-estradiol on MCF-7/2a breast cancer cells in the luciferase reporter gene assay (n = 3).** The procedure has been described elsewhere [34].
(TIF)

Figure S4 **Effect of the culture medium supplements (FCS, steroid depleted ct-FCS, phenol red) on the basal NPY Y_1_R expression by MCF-7 (L) cells.** All values (%) are related to the Y_1_R expression in the control experiment (100%, dashed line; stimulation with 1 nM 17β-estradiol in phenol red-free DMEM). Significance: *p<0.01 compared with DMEM plus ct-FCS, **p<0.01 compared with EMEM plus ct-FCS (n = 4 in all experiments).
(TIF)

Figure S5 **Masson-Goldner stained cryosections of MCF-7 (L) xenografts.** A: Control tumor C2, grown in nude mice substituted with estradiol. B: Tumor T2 from tamoxifen treated nude mice.
(TIF)
