# Supplementary figures and images for: The Neuropeptide Y Y1 Receptor: A Diagnostic Marker? Expression in MCF-7 Breast Cancer Cells Is Down-Regulated by Antiestrogens In Vitro and in Xenografts
Source: PLoS One. 2012 Dec 7;7(12):e51032. doi: 10.1371/journal.pone.0051032 (PMC3517602; doi:10.1371/journal.pone.0051032)

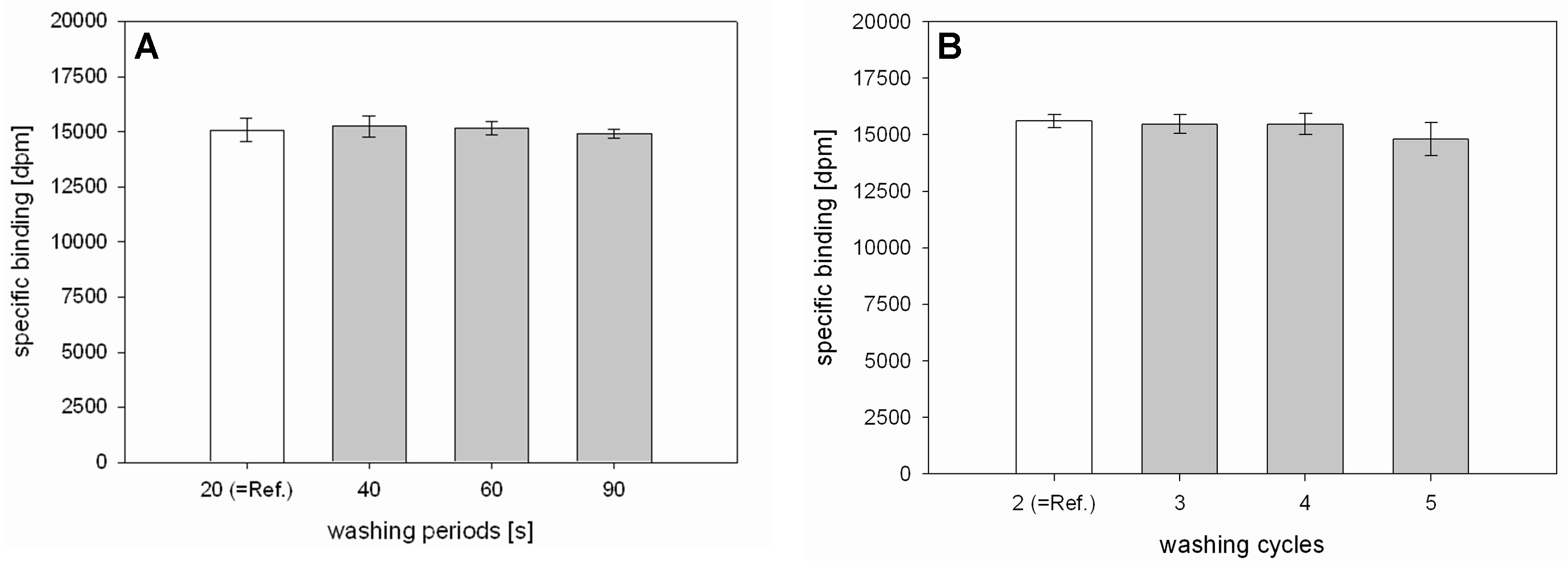

Supplement: Figure S1 — Specific binding of [3H]-UR-MK114 in dpm after varying washing conditions. (A) twice 20, 40, 60 and 90 s and (B) 2×, 3×, 4× and 5× 20 s; means ± S.E.M, n = 6. The experiments were performed to check for the dissociation of [3H]-UR-MK114 under the washing conditions applied in the radioligand binding assay. Basically, the experiments were conducted as already described in this paper and in [19]. Total binding was assessed with 12 nM of [3H]-UR-MK114, unspecific binding with radioligand (12 nM) plus a 300-fold excess of pNPY, all after an incubation time of 20 min at room temperature. A standard washing procedure of twice 20 s with ice cold buffer was set as reference. Then, conditions were varied in time and cycles, i.e. washing occurred at twice 40, 60 and 90 s as well as 3 times, 4 times and 5 times 20 s, all with n = 6. Under all washing conditions the specific binding was stable and only a negligible drop was observed with the longest period or the highest cycles. (TIF) [file pone.0051032.s001.tif]

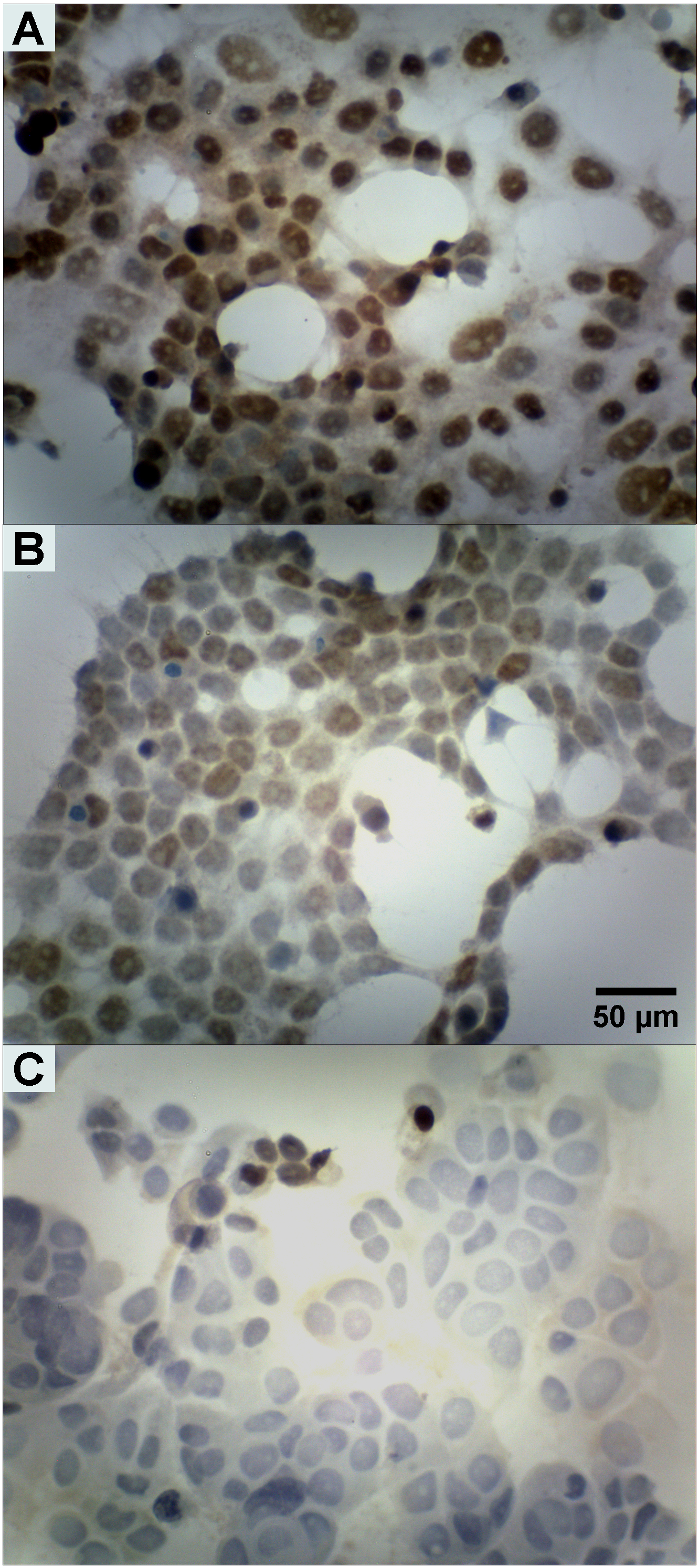

Supplement: Figure S2 — Immunocytochemical detection of the ERα expressed in different MCF-7 breast cancer cell variants according to the peroxidise/antiperoxidase method after paraformaldehyde fixation. Primary anti-human ER antibody clone 6F11 (LifeSpan BioSciences, Seattle, USA) using Ventana immunostainer (Ventana Medical Systems, Tucson, USA). MCF-7 cell with (A) high, (B) medium, and (C) low ER expression. (TIF) [file pone.0051032.s002.tif]

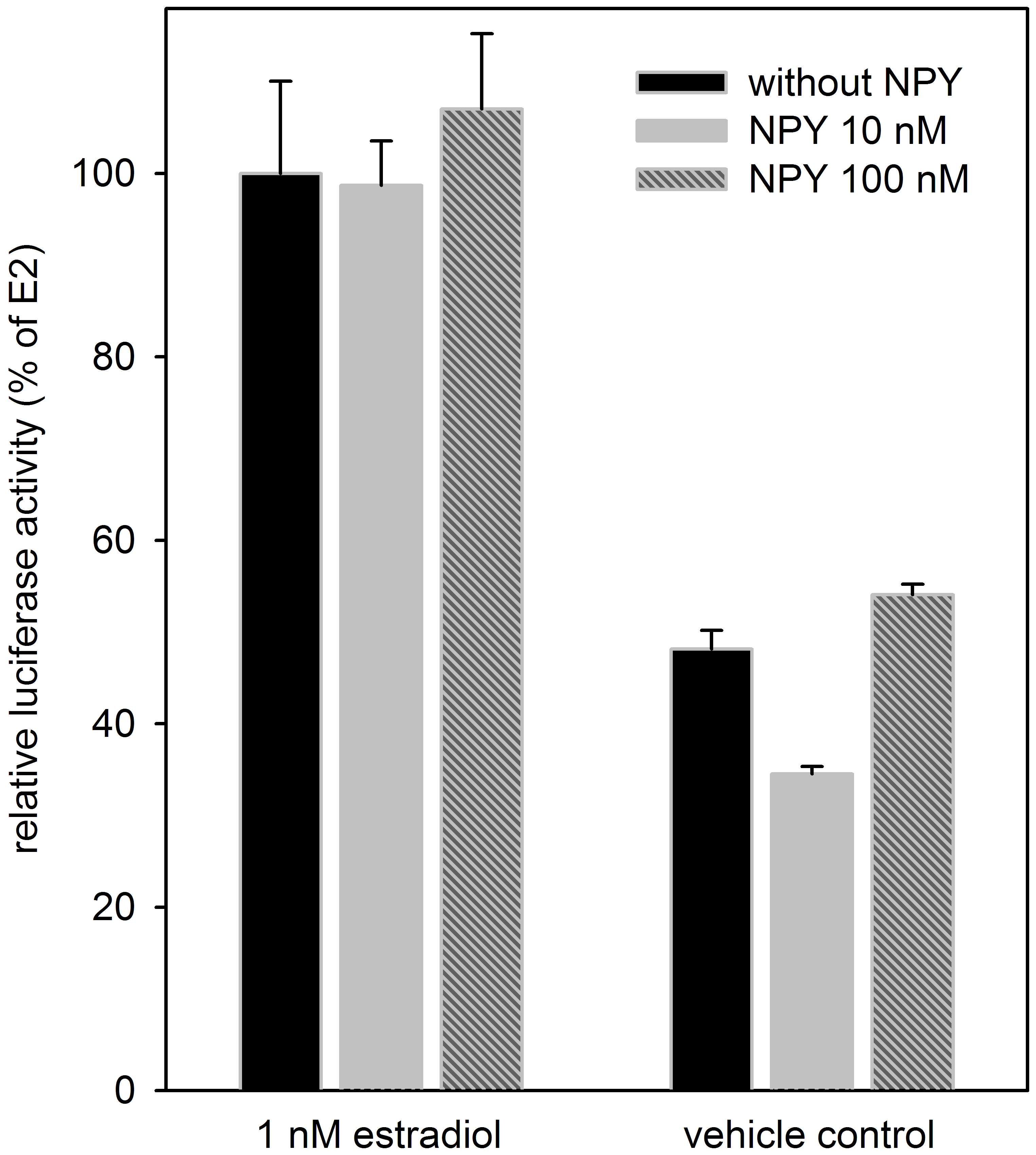

Supplement: Figure S3 — Effect of pNPY on the relative estrogenic activity of 17β-estradiol on MCF-7/2a breast cancer cells in the luciferase reporter gene assay (n = 3). The procedure has been described elsewhere [34]. (TIF) [file pone.0051032.s003.tif]

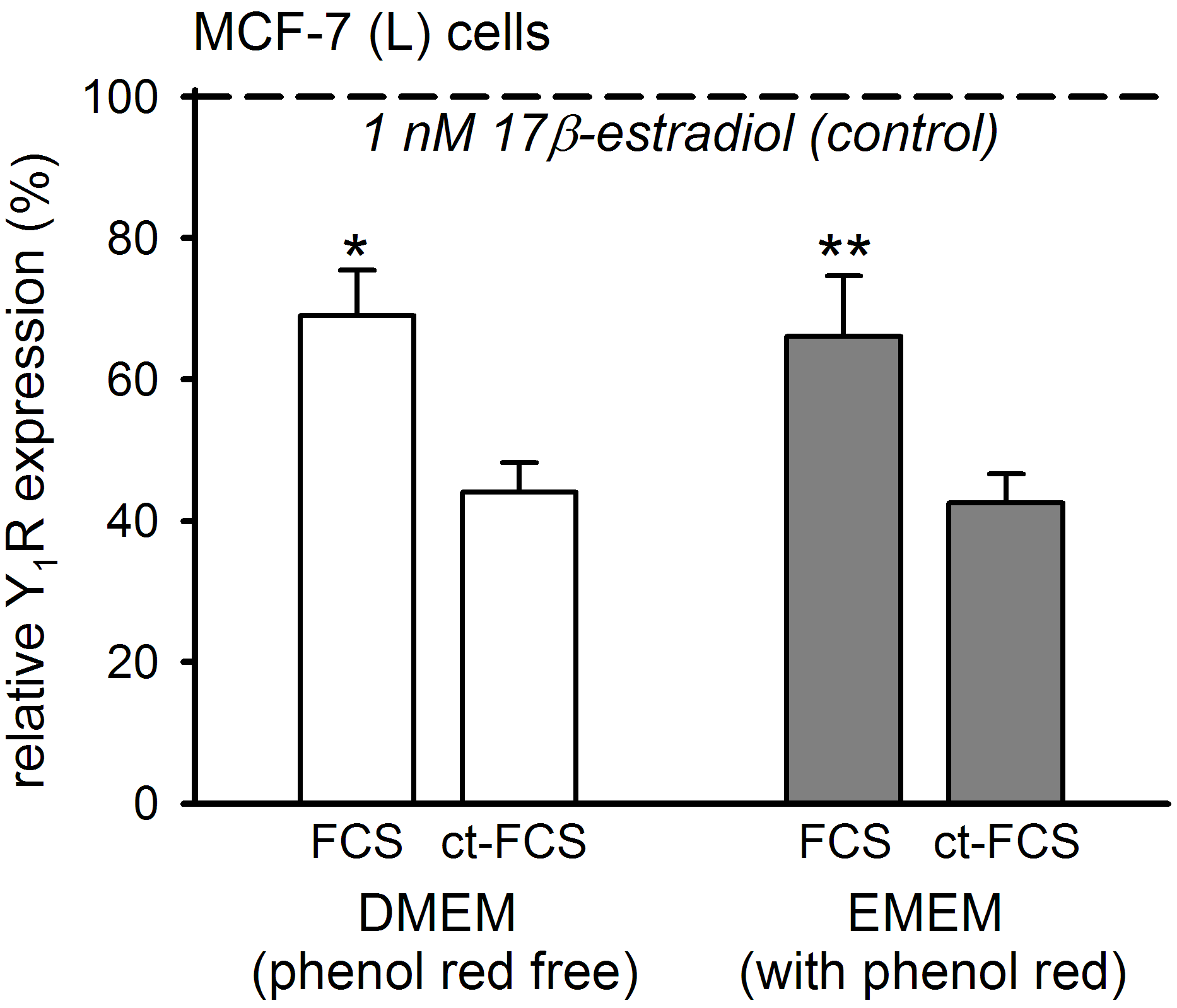

Supplement: Figure S4 — Effect of the culture medium supplements (FCS, steroid depleted ct-FCS, phenol red) on the basal NPY Y1R expression by MCF-7 (L) cells. All values (%) are related to the Y1R expression in the control experiment (100%, dashed line; stimulation with 1 nM 17β-estradiol in phenol red-free DMEM). Significance: *p<0.01 compared with DMEM plus ct-FCS, **p<0.01 compared with EMEM plus ct-FCS (n = 4 in all experiments). (TIF) [file pone.0051032.s004.tif]

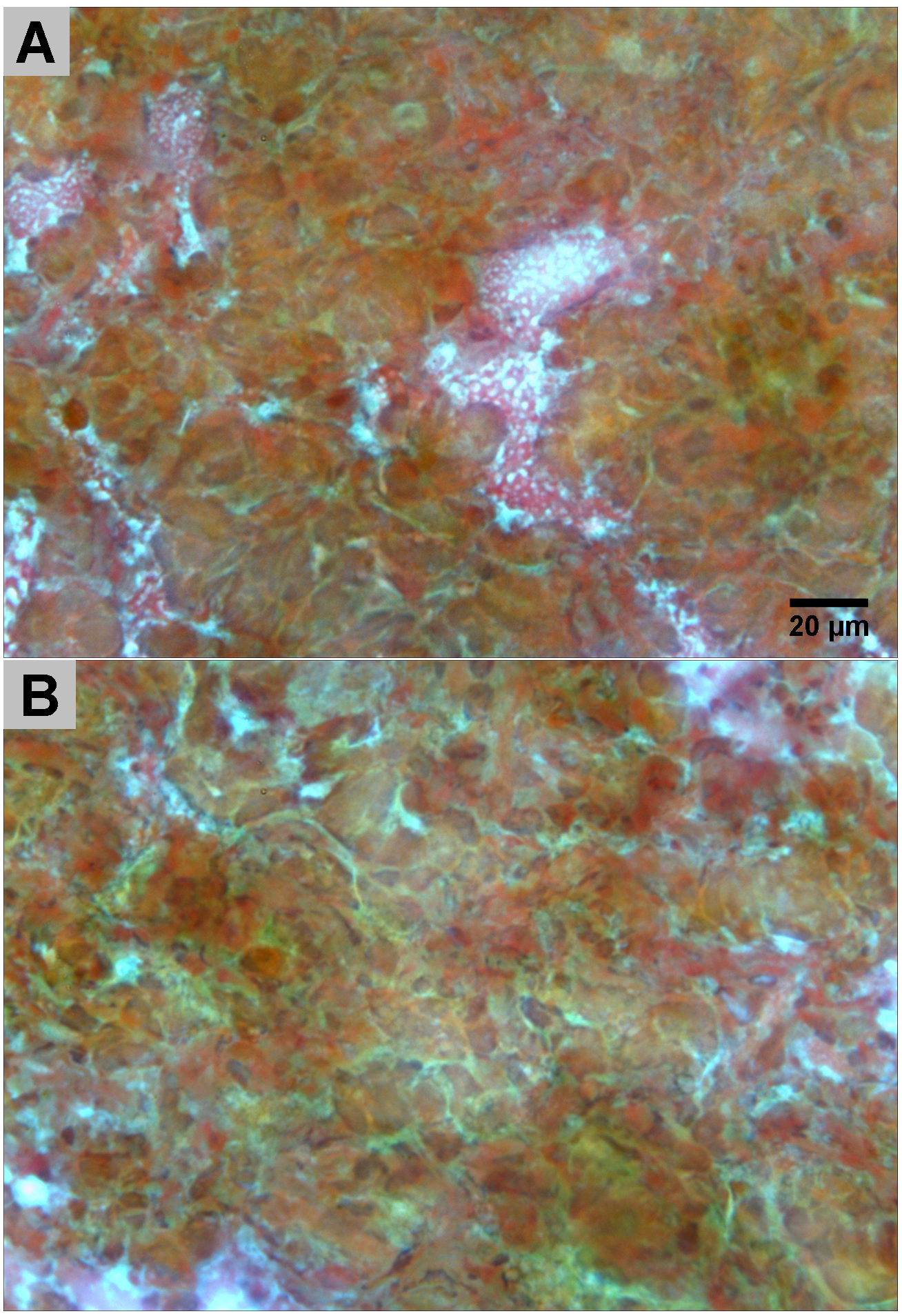

Supplement: Figure S5 — Masson-Goldner stained cryosections of MCF-7 (L) xenografts. A: Control tumor C2, grown in nude mice substituted with estradiol. B: Tumor T2 from tamoxifen treated nude mice. (TIF) [file pone.0051032.s005.tif]
